# On the robustness and replicability of the moderating influence of willpower mindset on the ego-depletion effect: existing evidence is weak at best

**DOI:** 10.3389/fpsyg.2023.1208299

**Published:** 2023-06-15

**Authors:** Akira Miyake, Nicholas P. Carruth

**Affiliations:** ^1^Department of Psychology and Neuroscience, University of Colorado Boulder, Boulder, CO, United States; ^2^Department of Psychology, DePaul University, Chicago, IL, United States

**Keywords:** ego depletion, implicit theories of willpower, willpower beliefs, self-control, replicability

## Introduction

Job et al. (2010; hereafter JDW) proposed that one's susceptibility to the ego-depletion effect—a performance decrement on a self-control task after performing on another self-control task—depends on one's implicit beliefs or mindset about whether willpower is limited or not (hereafter *willpower mindset*). Specifically, JDW demonstrated that only individuals holding a *limited*-willpower mindset exhibit a significant ego-depletion effect, a finding often regarded as a compelling refutation of a dominant resource-based account of the ego-depletion effect, the strength model (Baumeister et al., 2007). Berkman (2020) even called JDW's finding “the biggest bombshell” against the strength model.

Although we agree that the strength model has various theoretical problems (Lurquin and Miyake, 2017), JDW's willpower-mindset account also has major problems. Here, we first describe statistical problems that cast doubts on the robustness of JDW's original finding. We then present a review of existing ego-depletion studies we conducted to assess its replicability. We conclude by discussing additional major challenges surrounding JDW's account.

## Statistical problems

The statistical evidence for JDW's original finding (Study 1) is on shaky ground. JDW used a letter-cancelation (*e*-crossing) task as the depletion task and a Stroop task as the outcome task (*N* = 60). They analyzed the data using logistic hierarchical linear models (HLMs), with binary-coded accuracy on each Stroop incongruent trial (24 trials) as the dependent variable, and reported a significant interaction between the experimental manipulation (depletion vs. control) and willpower mindset: “*t*_(1433)_ = 3.88, *p* < 0.01” (p. 1688).

The reported degrees of freedom (1433), however, suggest that the model was likely misspecified, failing to properly nest trials under participants and thereby treating all 1440 trials—24 trials × 60 subjects—as independent observations (Schimmack, 2016). Their model also included three theoretically unmotivated covariates [trial order, reaction time (RT), and age], which may have increased the odds for false-positive results (Simmons et al., 2011).

JDW's subsequent studies analyzed the data differently, even when Stroop was the outcome task. Job et al. (2013) Experiment 1 (*N* = 87) used standard regressions, with Stroop interference in RTs (incongruent-congruent differences) as the dependent measure and age and lab rooms as covariates, and demonstrated depletion-reducing benefits of glucose consumption only among individuals holding a limited-willpower mindset. Savani and Job (2017) Study 3 used HLMs (properly nesting trials under subjects), with log-transformed RTs as the dependent measure and age and SES as covariates, and demonstrated a *reverse* ego-depletion effect (*better* Stroop performance under depletion) only among participants from India (*n* = 56) who collectively believed that exerting self-control is energizing (*p* = 0.045).

Such substantial data-analytic differences across studies raise doubts on the robustness of JDW's original finding, a concern exacerbated by our own failure to replicate the moderation effect in a preregistered study (*N* = 187; Carruth et al., 2023). To further assess the replicability of JDW's finding, we reviewed the existing literature.

## Review of existing literature

We conducted a literature search on Google Scholar in January 2023. We first identified all articles that cited the JDW study and then excluded (a) review/theoretical papers without any data and (b) research papers that did not report any ego-depletion studies. We examined the remaining papers to identify studies that administered JDW's willpower-mindset measure and tested its moderating influence on ego depletion. We included only studies treating willpower mindset as an individual-differences variable; those that experimentally manipulated willpower mindset were excluded (e.g., JDW's Study 2; Vohs et al., 2012, Experiment 1).

Thirteen independent studies (from 11 papers) met our inclusion criteria. We did not conduct a formal meta-analysis because different methods used to analyze the data in these studies (e.g., HLM, standard regressions)—some including covariates—made it difficult to cleanly derive a common effect-size metric for the hypothesized interaction effect without having access to the raw data.

We instead classified the 13 studies into two categories—those that observed moderating influences (Category A) and those that did not (Category B)—and examined whether they differed systematically. For this purpose, we coded the following variables: (a) final sample size; (b) data-analytic method; (c) timing of mindset measurement; (d) the study's primary focus; (e) significance of the overall ego-depletion effect; (f) significance of the moderation effect; (g) preregistration; and (h) data availability. The results are summarized in Table 1.

**Table 1:** Summary of the existing studies testing the moderating influence of willpower mindset on the ego-depletion effect.

**References**	**(a) Final sample size (# of participating labs)**	**(b) Analysis method used to test moderation**	**(c) The timing of mindset measure**	**(d) Is primary focus the moderation effect?**	**(e) Overall ego depletion significant?**	**(f) Moderation effect significant?**	**(g) Preregistered?**	**(h) Data shared publicly?**	**Notes**
**Category A: Studies that reported a moderating influence of willpower mindset on the ego-depletion effect**									
Chow et al. (2015) Experiment 3	*N* = 126	Regression	Beginning	Yes	Yes	Yes	No	No	
Dang et al. (2021)	*N* = 1,775 (*k* = 12)	Meta-analysis across labs	Beginning	No	Yes	Yes? (but only for a secondary DV)	Yes	Yes	The moderation was significant for a secondary RT measure, but not for the primary accuracy measure; it also disappeared when chance-level performance was excluded.
Job et al. (2010) Study 1	*N* = 60	Logistic HLM	Beginning	Yes	Yes?	Yes?	No	No	As noted in the main text, their HLMs appear to be misspecified and included *post-hoc*, theoretically unmotivated covariates.
Singh and Göritz (2019) Study 1	*N* = 115	Regression	Beginning	Yes	Yes?	Yes? (but in the opposite direction)	No	No	Willpower mindset moderated the ego-depletion effect, but the effect was stronger for those holding a nonlimited-willpower mindset. Because the journal bans the reporting of *p*-values, it is not clear whether this moderation effect was significant.
Singh and Göritz (2019) Study 2	*N* = 633	Regression	Beginning	Yes	Yes?	Yes? (but in the opposite direction)	No	No	In the initial analysis, there was no moderation effect, but, when mood (a hypothesized moderator) was added to the regression, the moderation effect emerged. Again, it was the opposite of what JDW found (i.e., more depletion for unlimited-willpower mindset). No *p*-values are reported due to the journal's policy.
**Category B: Studies that Reported No Significant Moderation Effect**									
Calvillo et al. (2022)	*N* = 192	Repeated-measure ANCOVA	End	No	Yes?	No	Yes	Yes	Depletion was manipulated within-subjects in this study. The overall ego-depletion effect was significant in an exploratory analysis that removed subjects who performed below-chance on the outcome task.
Carruth et al. (2023)	*N* = 187	Repeated-measure ANOVA and HLM	Beginning	Yes	No	No	Yes	Yes	A replication of JDW's Study 1. Tried different ways to analyze data to address statistical issues we noted in the main text.
Dang et al. (2017)	*N* = 176	Regression	Beginning	No	Yes	No	Yes	Yes	This study was the basis for the Dang et al. (2021) preregistered multilab study.
Garrison et al. (2019) Experiment 1	*N* = 657	HLM	End	No	Yes	No	Yes	Yes	
Garrison et al. (2019) Experiment 2	*N* = 359	HLM	End	No	Yes	No	Yes	Yes	
Singh and Göritz (2018)	*N* = 1,385	Regression	Beginning	No	No	No	No	Yes	
Vohs et al. (2021)	*N* = 3,531 (*k* = 36)	HLM	End	No	Yes?	No	Yes	Yes	The overall ego-depletion effect was significant only in an exploratory analysis based on the full sample, but not in the preregistered analysis.
Wenzel et al. (2019) Study 1	*N* = 187	HLM	Between Sessions	No	Yes	No	No	No	Depletion was manipulated within-subjects in this study.

This table includes only studies that administered Job et al. (2010) willpower mindset measure and tested its moderating influence on the ego-depletion effect using behavioral measures and comparing the deletion and control (no-depletion) conditions, just like JDW's Study 1. Studies that assessed the ego-depletion effect using self-reports without including depletion and control conditions are excluded. A small number of studies that manipulated subjects' willpower mindset experimentally (as opposed to treating it as an individual differences variable) are also not included here (e.g., JDW, Studies 2 and 3). DV, dependent variable; RT, reaction time; ANOVA, analysis of variance; ANCOVA, analysis of covariance; HLM, hierarchical linear modeling.

Five studies reported the moderating influence of willpower mindset on ego depletion (Category A). As explained in the table, however, the evidence in 4 of the 5 studies was ambiguous (including JDW's original study). Particularly noteworthy are two studies by Singh and Göritz (2019), both reporting the *opposite* moderation effect (greater depletion for those holding an *unlimited*-willpower mindset). Only one study (Chow et al., 2015) seems to have demonstrated the hypothesized moderation effect unambiguously.

Eight studies reported no significant moderation effect (Category B), even though the Category B studies collectively had greater sample sizes (mean *N* = 834.3; median *N* = 275.5) than the Category A studies (mean *N* = 464.5; median *N* = 96.5). Additionally, the hypothesized moderation effect did not seem to depend on the statistical significance of the overall ego-depletion effect: Although all 5 Category A studies reported some evidence for it, so did the majority of the Category B studies (6 out of 8).

The two categories of studies differed in several intriguing ways, however. First, all 5 studies that used HLMs and properly nested trials under participants failed to observe the moderation effect. Thus, it would be informative to know whether JDW's original moderation effect would still be significant if their data were analyzed with proper nesting.

Second, all Category A studies administered the willpower-mindset measure *before* the depletion manipulation. Administering willpower-mindset and other self-control-related measures early has the advantage of avoiding questionnaire responses being influenced by the depletion manipulation, but it runs the risk of the questionnaire items influencing participants' subsequent behaviors. Interestingly, all 5 studies that administered the mindset questionnaire at the end and thereby minimized such potential priming influences failed to observe the moderation effect.

Third, testing the moderating influence of willpower mindset on ego depletion was the primary focus in 4 of the 5 Category A studies, but in only 1 of the 8 Category B studies (the other studies focused primarily on the replicability of the ego-depletion effect itself; willpower mindset was included as one of the few potential moderators and did not receive much discussion). One way to interpret this discrepancy is in terms of publication bias: If the study's main purpose is to test the moderation effect, publishing a nonsignificant result may be more difficult, hence resulting in few such studies in Category B.

Finally, almost all Category B studies were preregistered (minimizing the likelihood of *p*-hacking) and have publicly shared the data (enabling other researchers to verify the results and reanalyze the data), but not for the Category A studies, with one notable exception of the Dang et al. (2021) study. The evidence reported in that preregistered multilab study was ambiguous, however: The moderation effect was significant (*p* = 0.039) only for their secondary dependent measure (RTs for the antisaccade task), and the effect became nonsignificant when the analyses excluded subjects who likely failed to understand the task requirements and performed the outcome task at chance level.

This brief review is limited in two important ways. First, we did not conduct a formal meta-analysis and hence cannot provide an aggregate effect-size estimate for the target interaction term or quantitative tests of the moderating variables we coded for each study. Second, this review was restricted to published studies. Accordingly, it is important to keep in mind that a meta-analysis that includes unpublished studies may yield different results. It would therefore be worthwhile to conduct such a meta-analysis, especially based on raw data so that effect sizes for the target interaction term can be estimated more equivalently across studies.

Despite these limitations, our review showed that the existing evidence for JDW's original finding is weak at best: No large-sample preregistered study currently exists that yielded robust evidence for the hypothesized moderation effect for the primary dependent measure. The finding that only one of the 13 studies yielded an unambiguous moderation effect in the predicted direction casts seriously questions the replicability of JDW's original finding.

## Additional challenges

JDW's willpower-mindset account of ego depletion also faces other challenges. First, their account assumes that the ego-depletion effect is “real” at least among some individuals. However, both meta-analyses (Carter and McCullough, 2014; Carter et al., 2015) and large-scale multilab studies (Hagger et al., 2016; Vohs et al., 2021) have seriously challenged the replicability of the ego-depletion effect itself (Friese et al., 2019). Moreover, self-reports from researchers who have conducted ego-depletion studies revealed an alarming rate of prior engagement in questionable research practices, especially selective reporting of outcomes (Wolff et al., 2018). Thus, it is far from clear whether the ego-depletion effect is real.

Even if this controversial effect is real, its overall effect size would likely be small. Three preregistered multilab studies of ego depletion, for example, yielded effect-size estimates (Cohen's *d*) of only 0.04 (Hagger et al., 2016), 0.08 (Vohs et al., 2021), and 0.10 (Dang et al., 2021), respectively. Detecting a difference between two independent groups with a 0.80 power requires >3,000 participants when the effect size is 0.10. Given that detecting a statistical moderation effect involving individual-differences variables has been known to be difficult (McClelland and Judd, 1993), replicating JDW's original finding (*N* = 60) with sufficient power would present a major challenge in future research, requiring much larger sample sizes than used before in ego-depletion studies.

JDW's account is also lacking theoretically (Bertrams, 2020). Although not explicitly stated in the original article, Job's preferred explanation is motivational: Performing a demanding self-control task reduces one's motivation level, especially for those holding a limited-willpower mindset, thus making them more willing to “slack off” on a subsequent task (see Carruth et al., 2023, for further elaboration). Though plausible, this motivational account of ego depletion is not precise enough to allow other researchers to unambiguously derive testable (falsifiable) predictions, a point we also raised against the strength model (Lurquin and Miyake, 2017). Without better specifying under what circumstances individuals with a limited-willpower mindset decide to “slack off,” it is difficult to unambiguously predict whether willpower mindset has a moderating influence on ego depletion in a given study.

Equally challenging is the observation that the existing studies showed three different patterns: (a) stronger depletion for *limited*-willpower mindset; (b) stronger depletion for *unlimited*-willpower mindset; and (c) no effect of willpower mindset. Given that JDW's account is unidirectional (limited-willpower mindset → greater depletion effects), it cannot explain the opposite pattern reported by Singh and Göritz (2019). Considering that Clarkson et al. (2016) and Vohs et al. (2012) (Experiment 1) demonstrated that an experimentally induced *unlimited*-willpower mindset can lead to *worse* self-control performance under some circumstances, one cannot simply dismiss the Singh and Göritz (2019) finding as a fluke. If (a) and (b) are both real and reflect two different ways in which willpower mindset impacts participants' motivation for self-control, JDW's account clearly needs a further theoretical specification.

One possible way forward is to specify potential underlying moderators and mediators that can help reconcile patterns (a) and (b). In fact, Clarkson et al. (2016) proposed *(dis)fluency* (subjective ease/difficulty of retrieving or processing information) as the moderator (fluency promotes JDW's original pattern, whereas disfluency reverses it) and *perceived mental fatigue* as the mediator. It is an intriguing proposal, but, because Clarkson et al. (2016) did not test their account using an ego-depletion paradigm, it is unclear whether this account is readily applicable to the ego-depletion results reviewed here.

We believe that willpower mindset will likely remain a useful concept in self-control research (see Francis and Job, 2018, for a review). However, its application to ego depletion—the very phenomenon whose existence has been questioned—is not as compelling as often portrayed in the literature: The moderating influence of willpower mindset on ego depletion is weak at best, starting with JDW's original study. Until more compelling evidence is provided and a more precise model is developed, JDW's original finding must be treated with great caution.

## Author contributions

AM and NC jointly developed the ideas and the plan for the literature review presented in this article. NC conducted the literature search and produced an initial draft of the summary table for the literature review. AM checked the entries, edited the table, and produced an initial draft of the main text. Both contributed to the editing of the text.
